# Knowledge graph and machine learning-guided virtual screening prioritizes putative blockers of the PSMD11/Rpn6-EBOV glycoprotein interface

**DOI:** 10.3389/fphar.2026.1910440

**Published:** 2026-09-29

**Authors:** Yanshuang Wang, Yuanjian Li, Lin Zhu, Zhiguo Jiang, Qian Tang, Hainan Liu, Yong Hu, Zijing Liu, Ziyi Li, Xinwei Zhou, Xin Qi, Yinan Liao, Luya Hu, Yufeng Wang, Yue Liu, Ziqian An, Qincai Dong, Cheng Cao

**Affiliations:** 1 National Key Laboratory of Advanced Biotechnology, Academy of Military Medical Sciences, Beijing, China; 2 School of Life Sciences and Medical Engineering, Anhui University, Hefei, China

**Keywords:** ADMET, Co-IP/MS, EBOV GP, knowledge graph, machine learning, molecular docking, proteasome, PSMD11

## Abstract

**Introduction:**

Host-directed antiviral discovery requires a traceable route from host-factor identification to testable molecular hypotheses. This study developed such a workflow around a host factor associated with Ebola virus glycoprotein (EBOV GP).

**Methods:**

GFP-GP pulldown coupled with co-immunoprecipitation/mass spectrometry was used to identify GP-associated host candidates. A PSMD11/Rpn6-centred workflow integrated knowledge-graph inference, ligand-only machine learning, ADMETlab profiling and interface-guided docking. Candidate prioritization was assessed through score-weight sensitivity analysis, applicability-domain assessment and docking controls addressing seed reproducibility, search convergence, whole-receptor context and macrocycle ring-conformer sensitivity. Docking scores were contextualized against a matched 59-compound library.

**Results:**

The analysis recovered 424 GP-pulldown-specific host proteins and nominated PSMD11/Rpn6 as a proteostasis anchor. The tier-gated knowledge-graph candidate membership was invariant across the tested weight perturbations, with Spearman rho = 0.998 under equal weighting. The selected ExtraTrees model achieved an AUC of 0.912 for the Ki endpoint. Applicability-domain assessment and ADMET profiling differentiated candidate confidence and predicted liabilities. Docking reproducibility checks yielded a maximum standard deviation of 0.05 kcal/mol for conformationally restricted ligands, while macrocyclic candidates showed greater protocol-dependent variability. Rule-based triage integrating upstream evidence, ADMET burden, chemotype diversity and mechanistic coverage returned five candidates: betamethasone, bafilomycin A1, everolimus, pitavastatin and CHEMBL1410015.

**Discussion:**

The workflow links a proteomic host-factor signal to a compact set of chemically diverse hypotheses with explicit evidence sources and limitations. The candidates remain hypotheses for experimental assessment; direct target engagement, interface blockade and antiviral activity are not established by the present study.

## 1 Introduction

Ebola virus disease remains a major cause of high-fatality viral haemorrhagic fever, first recognized as a distinct filovirus disease in the 1970s and repeatedly associated with severe regional outbreaks thereafter (Feldmann and Geisbert, 2011). Although rVSV-vectored vaccination, monoclonal-antibody therapy and improved supportive care have strengthened outbreak responses, the clinical and public-health management of EBOV infection is still constrained by cryptic transmission, narrow treatment windows and the complexity of virus-host interactions (Henao-Restrepo et al., 2017; Mulangu et al., 2019). EBOV GP is the dominant viral envelope glycoprotein and mediates particle attachment, endocytosis, endosomal proteolytic priming, Niemann-Pick C1 (NPC1) engagement and membrane fusion (Kuroda et al., 2020; Bortz et al., 2020; Lane and Ekins, 2020; Plewe et al., 2020; Gaisina et al., 2020; Chandran et al., 2005; Carette et al., 2011; Kuroda et al., 2015; Gong et al., 2016; Simmons et al., 2016). It is therefore a central target of neutralizing antibodies, entry inhibitors and host-factor intervention strategies.

Previous work has defined many of the core steps in GP-mediated entry. Proteolytic processing of GP within the endosomal-lysosomal pathway exposes receptor-binding determinants that allow NPC1 engagement and subsequent membrane fusion. NPC1 functions as a well-established intracellular receptor that links GP conformational priming to the fusion step of EBOV entry (Gong et al., 2016; Bornholdt et al., 2016; Ahmad et al., 2023). Its identification as an essential entry factor also provides a conceptual precedent for host-directed EBOV intervention, in which host proteins required for GP-mediated infection can be used as mechanistic anchors for antiviral discovery. Additional host factors, including TIM-1, the HOPS complex, PIKFYVE, GNPTAB, PIK3C3, SLC39A9 and glycosylation-related pathways have been supported by genetic screens, structural studies or infection models (Chandran et al., 2005; Carette et al., 2011; Kuroda et al., 2015; Gong et al., 2016; Simmons et al., 2016; Flint et al., 2019; Gong et al., 2024). Soluble GP, GP-induced endothelial signalling, GP conformational stability and GP glycosylation may also influence viral pathogenicity or entry efficiency (Bortz et al., 2020; Moni et al., 2022; Furuyama et al., 2021; Bagdonaite et al., 2024). Together, these findings indicate that GP-associated host dependence extends beyond a single receptor-binding event and involves endocytic trafficking, glycoprotein processing, membrane fusion, cellular stress responses and immune regulation.

Compared with entry receptors and endocytic trafficking factors, GP-associated proteostasis nodes have been less systematically exploited for candidate molecule discovery. The ubiquitin-proteasome system (UPS), endoplasmic-reticulum stress, the unfolded protein response and broader proteostasis machinery are recurrent host-dependence or host-restriction contexts during viral infection; proteostasis here refers to the integrated cellular network that maintains protein folding, trafficking, assembly and degradation capacity (Valerdi et al., 2021; Prasad and Greber, 2021; Balch et al., 2008). Viruses can reshape protein stability, replication-complex assembly, immune evasion and entry or egress through ubiquitination, deubiquitination, proteasomal degradation, folding burden and stress signal (Valerdi et al., 2021; Prasad and Greber, 2021; Lun et al., 2021; Umthong et al., 2021; Song et al., 2021; Abad et al., 2023). For enveloped viruses such as EBOV, the synthesis, glycosylation, folding and trafficking of large amounts of GP may itself impose a substantial burden on host quality-control systems. This provides a biological rationale for searching GP interactomes for proteostasis-associated host anchors.

PSMD11/Rpn6 is a structural and regulatory subunit of the 19S regulatory particle lid of the 26S proteasome. Structural studies indicate that PSMD11/Rpn6 contributes to stabilization of the interface between the 19S regulatory particle and the 20S core particle, while changes in PSMD11/Rpn6 expression or function can alter proteasome activity and proteostasis output (Pathare et al., 2012; Lander et al., 2012). Small-molecule probe and target-fishing studies further suggest that PSMD11/Rpn6 is chemically interrogable (Tian and Trader, 2020; Dai et al., 2022). Moreover, PSMD11/Rpn6 loss-of-function has been linked to impaired 26S proteasome assembly and increased interferon-response phenotypes, placing PSMD11/Rpn6 at a possible intersection between proteostasis and immune regulation (Deb et al., 2024). These observations provide a plausible basis for evaluating it as a proteostasis anchor in a tiered discovery workflow.

Host-directed antiviral discovery offers the potential advantage of targeting host dependencies that are not encoded by highly mutable viral genomes. However, this strategy faces two methodological difficulties. First, interactomics and genetic screens frequently generate large host-candidate lists, and without matched controls and explicit evidence stratification it is difficult to distinguish reproducible interactions from non-specific pulldown background, expression-burden effects or mechanistically relevant nodes. Second, host-targeted small molecules often carry liabilities related to broad targeting, cytotoxicity, immune modulation or pharmacokinetics; therefore, they should not be advanced on the basis of a single docking score, knowledge-graph rank or ligand-only machine-learning output. Knowledge graphs and graph-based drug-repurposing methods provide useful frameworks for integrating heterogeneous evidence (Zhu et al., 2020; Zhang et al., 2023), but they require experimental anchoring to avoid becoming generic database-driven inference.

We therefore adopted an experimental-anchor-first, computational-stratification-second strategy. Co-IP/MS was used to nominate GP-associated host signals; knowledge-graph inference was used to place candidates in a mechanistic network context; ligand-only machine learning was used to estimate relative bioactivity tendencies; ADMET profiling was used to constrain experimental risk; and interface docking was used to generate control-audited structural accommodation hypotheses. Using this framework, we asked whether PSMD11/Rpn6 can be nominated as a GP-pulldown-associated proteostasis candidate, whether PSMD11/Rpn6-centred computation can yield mechanistically interpretable and risk-stratified molecules, and which candidates are most suitable for testing GP-PSMD11/Rpn6 association, proteostasis-related phenotypes and compound perturbation effects.

## 2 Materials and methods

### 2.1 Co-IP/MS dataset and sample inclusion

HEK293 Co-IP/MS data served as the experimental anchor. Two matched anti-GFP pulldown samples from the same batch were included: F005448, which expressed a GFP-GP fusion protein and served as the GFP-GP bait sample, and F005449, which expressed the pEGFP-C1 GFP empty vector and served as the GFP-only negative control. Both samples were subjected to anti-GFP bead enrichment, tryptic digestion and LC-MS/MS analysis. This paired design was intended to distinguish GFP-GP-associated complex signals from GFP- and bead-associated background within a shared tag and pulldown context. Samples were included when they originated from the same HEK293 Co-IP/MS experiment, had clearly defined bait or control constructs, contained traceable Mascot protein- and peptide-level outputs suitable for UniProt-accession-level merging, and had been searched with identical database parameters. Samples from unrelated expression conditions, samples lacking traceable Mascot outputs, or samples that did not directly support the GFP-GP versus GFP-only comparison were excluded.

For orthogonal cell-level assessment of the GP-associated PSMD11/Rpn6 candidate, HeLa cells were co-transfected with GP-RFP and PSMD11-GFP or GFP-alone control. At 24–48 h after transfection, cells were washed twice with PBS, fixed with 4% paraformaldehyde in PBS for 15–20 min at room temperature, washed three times, permeabilized with 0.2%–0.5% Triton X-100 in PBS for 10–15 min, and blocked with 5% bovine serum albumin or 10% normal goat serum for 1 h. Coverslips were mounted with ProLong Gold antifade medium containing DAPI. Images were acquired using a confocal laser-scanning microscope with 488-nm, 561-nm and 405-nm excitation for GFP, RFP and DAPI, respectively, using a 60x or 63x oil-immersion objective. Channels were acquired sequentially to minimize crosstalk. The analysis assessed spatial overlap of GP-RFP and PSMD11-GFP; it was not used to infer direct binding.

### 2.2 LC-MS/MS search, preprocessing and quality control

Raw LC-MS/MS data were searched with Mascot against SwissProt, using established database-search logic for probability-based protein identification and the curated UniProtKB/Swiss-Prot protein sequence resource (Perkins et al., 1999; The UniProt Consortium, 2017; Boutet et al., 2007). Search settings included trypsin specificity, a maximum of one missed cleavage, cysteine carbamidomethylation as a fixed modification, methionine oxidation as a variable modification, 10 ppm precursor mass tolerance, 0.6 Da fragment mass tolerance, monoisotopic mass values and a significance threshold of p < 0.05. Each sample contained 30,639 MS/MS queries. The use of identical database versions, digestion rules, modification settings and mass tolerances ensured that the two samples were compared within the same search space. Mascot protein-level reports were merged at the UniProt accession level. Proteins not detected in a sample were assigned values of 0 for Mascot score, unique peptide count and sequence coverage only for presence/absence classification; these values were not interpreted as true abundance estimates. Proteins were categorized as GP-pulldown-only preliminary candidates, shared/non-specific proteins, or control-specific proteins according to their detection pattern in F005448 and F005449. PSMD11/Rpn6 peptide-level evidence was extracted from Mascot peptide exports and protein reports and verified by exact matching to the canonical sequence (UniProt O00231). PSMD11/Rpn6 was considered eligible as a host anchor if it was detected in GFP-GP pulldown, absent from the matched GFP-only control and supported by peptide sequences matching the canonical PSMD11/Rpn6 sequence.

### 2.3 PSMD11/Rpn6-centred heterogeneous knowledge graph

A PSMD11/Rpn6-centred heterogeneous biomedical knowledge graph was constructed by integrating curated literature evidence, DRKG-derived compound-gene target edges, proteasome/proteostasis module annotations, viral host-interaction context, MS seed evidence and manually audited triples. Entity types included genes/proteins, compounds, pathways, diseases, viral contexts and literature-derived evidence nodes. Relation types included compound-target, host-machinery, proteasome-module, PSMD11/Rpn6-neighbourhood, shared-anchor transfer and embedding-supported completion candidates. Known PSMD11/Rpn6 probes, direct PSMD11/Rpn6 knowledge-graph evidence compounds and classical proteasome inhibitors were retained as audit or control references. The discovery queue was generated from inference-only candidates to avoid allowing known direct probes or canonical proteasome inhibitors to dominate exploratory ranking.

### 2.4 Knowledge-graph inference scoring

Candidate ranking used a predefined heuristic triage score rather than a model trained on an external validation set:

KG score=0.48×TransE_supported+0.20×PSMD11_neighbor+0.19×proteasome_module+0.13×shared_anchor_transfer−target_count_penalty

TransE_supported denotes embedding-supported completion evidence; PSMD11_neighbor denotes proximity to the PSMD11/Rpn6 neighbourhood; proteasome_module denotes association with proteasome or proteostasis modules; shared_anchor_transfer denotes indirect inference through shared host or mechanistic anchors; and target_count_penalty reduces the discovery priority of broad-target candidates. The final KG top20 used a rule-tiered queue, with ranks 1–12 retaining candidates with contextual PSMD11-side evidence and ranks 13–20 serving as TransE-only reserve candidates. These weights were fixed before scoring, so the queue is reproducible from the published formula and candidate nomination is governed by the staged tier rules rather than by the raw score. To make the effect of this choice explicit, KG score-weight sensitivity was assessed under equal component weights, single-component perturbations (×0.5-×1.5), target-count-penalty perturbations and random Dirichlet weight perturbations (Supplementary Figure S1; Supplementary Table S6).

### 2.5 Ligand-only machine learning affinity filtering

Training data were derived from PDBbind protein-ligand binding records and split into IC50, Kd and Ki endpoint tables. The KG top20 candidates were subjected to ligand-only machine-learning filtering using canonical SMILES as input. Compounds that could not be parsed into valid molecular objects or feature matrices were excluded. Molecular features consisted of Morgan/ECFP4 2048-bit fingerprints and ten RDKit physicochemical descriptors: molecular weight, MolLogP, topological polar surface area, hydrogen-bond donor count, hydrogen-bond acceptor count, rotatable-bond count, aromatic-ring count, heteroatom count, fraction Csp3 and heavy-atom count (Rogers and Hahn, 2010; Landrum, 2013; Bento et al., 2020). Binary classification heads were trained separately for IC50, Kd and Ki endpoints using predefined train/test/validation splits. The IC50 dataset contained 4,301 samples (2,567 training, 868 test and 866 validation samples); the Kd dataset contained 6,466 samples (3,876 training, 1,298 test and 1,292 validation samples); and the Ki dataset contained 3,434 samples (2,054 training, 690 test and 690 validation samples). Extra Trees, Random Forest, Logistic Regression, Gradient Boosting and Linear SVM models were compared using AUC, AUPR, accuracy, F1 score, Matthews correlation coefficient and confusion matrices (Geurts et al., 2006; Breiman, 2001; Hosmer et al., 2013; Friedman, 2001; Jakkula, 2006; Rainio et al., 2024).

A composite machine-learning score was defined as:

ML score=0.70×Ki+0.20×IC50+0.10×Kd

Ki was weighted most strongly because it is more directly related to binding or inhibition constants and because the Ki classifier showed the best internal performance in this analysis. IC50 was used as a functional-activity support signal and Kd as a reference signal. The ML score quantifies relative activity tendency within the available bioactivity feature space, which is the axis this layer is designed to contribute. The endpoint weights were likewise fixed before scoring, and endpoint-weight sensitivity was assessed under equal weights, single-endpoint perturbations and Dirichlet perturbations (Supplementary Figure S2; Supplementary Table S6). To define the chemical space in which each score is supported, the applicability domain of the ligand-only models was audited against the endpoint-specific training ligands using ECFP4 nearest-neighbour Tanimoto similarity together with RDKit descriptor-range checks, and each candidate was classified as inside or well supported, borderline or mixed, or extrapolative (Supplementary Figure S3; Supplementary Table S7).

### 2.6 ADMET risk stratification

ML top12 candidates were profiled with ADMETLab 3.0, an online ADMET prediction platform for absorption, distribution, metabolism, excretion and toxicity assessment (Fu et al., 2024). Risk categories included hERG/QT liability, drug-induced liver injury, Ames/genotoxicity, organ toxicity, CYP-related drug-drug interaction, assay interference, physicochemical out-of-range properties, absorption and solubility. Because EBOV persists in immune-privileged central-nervous-system sites while acute replication is systemic, both predicted CNS access and predicted peripheral restriction are informative, so the endpoint was recorded for all candidates rather than applied as a pass/fail criterion (Supplementary Figure S4; Supplementary Table S7). A local overall_admet_risk_proxy was derived from ADMETLab outputs to summarize relative risk burden within this candidate set. This proxy supports within-cohort prioritization and is reported alongside the individual ADMETLab endpoints. The structural follow-up shortlist was then selected by explicit triage rules: candidates with broad ADMET red flags were shifted to comparator/control roles; the remaining candidates had to retain at least one upstream support signal from KG or ML; chemically or mechanistically redundant options were collapsed to one representative; and the final set was required to cover distinct, experimentally interpretable mechanisms while remaining compatible with interface docking. The final five candidates therefore constitute the structural follow-up shortlist produced by these staged rules, each entering with a named evidence source.

### 2.7 Interface definition and molecular docking

Structural follow-up focused on pockets near the predicted PSMD11/Rpn6 and EBOV GP interface, rather than on arbitrary sites in isolated proteins. PSMD11/Rpn6 (UniProt O00231) and EBOV GP (UniProt Q05320) were modelled as a protein complex using AlphaFold 3, which predicts biomolecular structures and interactions across protein, nucleic-acid and small-molecule contexts (Fu et al., 2024). All five AlphaFold 3 complex models returned ipTM 0.14–0.15, pTM 0.41 and fraction disordered 0.27–0.29, so the complex was used to define an interface-proximal search region and the resulting docking evidence was bounded by the four control experiments described below. The selected exploratory model was used to define GP-contacting residues on the PSMD11/Rpn6 side, and nearby pockets, grooves and shallow cavities were selected for docking-box placement. Preliminary docking was performed using AutoDock Vina (Trott and Olson, 2010). The search box was centred on the PSMD11/Rpn6-side interface pocket derived from the AlphaFold 3 complex model and expanded with padding to cover adjacent grooves and cavities near the predicted GP-contacting surface. The complete grid specification is now reported: box centre (−11.079, 5.480, −0.367) Å and box dimensions 40.14 × 25.64 × 43.68 Å (volume 44,952 Å^3^), with grid spacing 0.375 Å, exhaustiveness 8, energy_range 3 kcal/mol and num_modes 9; receptor and ligands were prepared as rigid-receptor PDBQT files and the per-run random seeds are listed in Supplementary Table S8. This box encloses 28.5% of receptor heavy atoms and 138 of 422 PSMD11/Rpn6 residues, defining a regional search volume around the predicted interface that covers the interface footprint together with its adjacent grooves and cavities. Docking poses were inspected in PyMOL to evaluate their spatial placement relative to the putative interface and to generate structural visualizations (DeLano, 2002).

To define the boundary conditions of the docking evidence, four control experiments were performed on the archived receptor and ligand files. First, each reported pose was re-docked with the archived configuration under five random seeds, and again at exhaustiveness 32, to quantify reproducibility and search convergence. Second, a whole-receptor blind-docking control (108 × 68 × 84 Å box covering the entire PSMD11/Rpn6 model) was run under five seeds to test whether non-interface pockets yield equal or better predicted scores. Third, because Bafilomycin A1 and Everolimus are macrocycles, both were re-prepared with a macrocycle-aware protocol in which ring bonds are broken with glue pseudo-atoms so that ring conformation is sampled during docking, and were additionally docked as a 20-member ETKDGv3 ring-conformer ensemble; these were compared against the rigid-ring preparation used originally. Fourth, the KG-scored candidate pool (59 ligands comprising the PSMD11-side target-transfer and TransE-completion tiers, with the published audit anchors and proteasome-inhibitor positive controls removed) was docked into the identical interface box to establish the score distribution expected for an arbitrary compound of this class in this volume. All control runs used AutoDock Vina 1.2.5; scripts, logs and per-run outputs are provided in the revision package (Supplementary Figures S5-S7; Supplementary Tables S8-S11).

## 3 Results

The initial GFP-GP pulldown signal was converted into an experimentally anchored candidate-discovery path (Figure 1). Co-IP/MS first tested whether the GP bait enriched a host-candidate space distinct from GFP-associated background. PSMD11/Rpn6 was then used as a proteostasis-related anchor to constrain knowledge graph inference, machine learning model, ADMET assessment and interface-focused docking.

**FIGURE 1:**
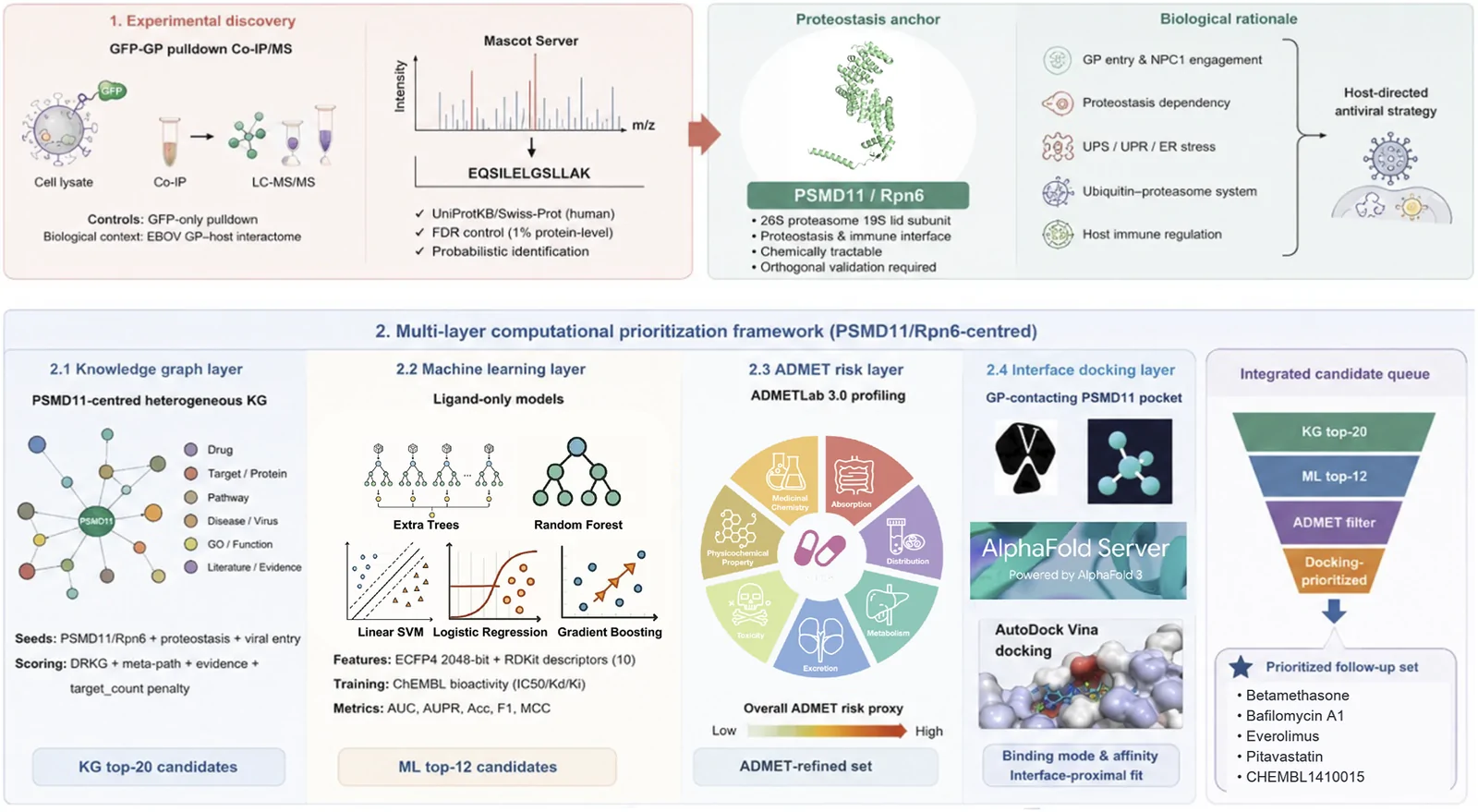
PSMD11/Rpn6-centred workflow for prioritizing EBOV GP-associated proteostasis candidates.

### 3.1 Co-IP/MS identifies PSMD11/Rpn6 as a GP-pulldown-associated proteostasis anchor

Co-IP/MS identified 1,286 distinct UniProt accessions across the GFP-GP pulldown and matched GFP-only control. The critical observation was selective rather than quantitative: PSMD11/Rpn6 fell within the GFP-GP-associated candidate space and was absent from the GFP-only background (Figure 2a). The overall classification of 424 GP-pulldown-only proteins, 641 shared proteins and 221 control-specific proteins defined the experimental background against which this signal was interpreted (Figure 2b). The PSMD11/Rpn6-specific evidence then localized the anchor within this background: the protein-level summary confirmed its detection pattern (Figure 2c), and peptide mapping placed the representative peptide EQSILELGSLLAK at residues 47–59 with a Mascot ion score of 33.9 (Figure 2d,e). PSMD11/Rpn6 was therefore advanced as a selectively detected proteasome subunit combining biological relevance to UPS/proteostasis and viral host-dependence contexts with prior chemical tractability established in PSMD11/Rpn6 small-molecule studies. This evidence nominates PSMD11/Rpn6 as a GP-associated proteostasis anchor for downstream prioritization.

**FIGURE 2:**
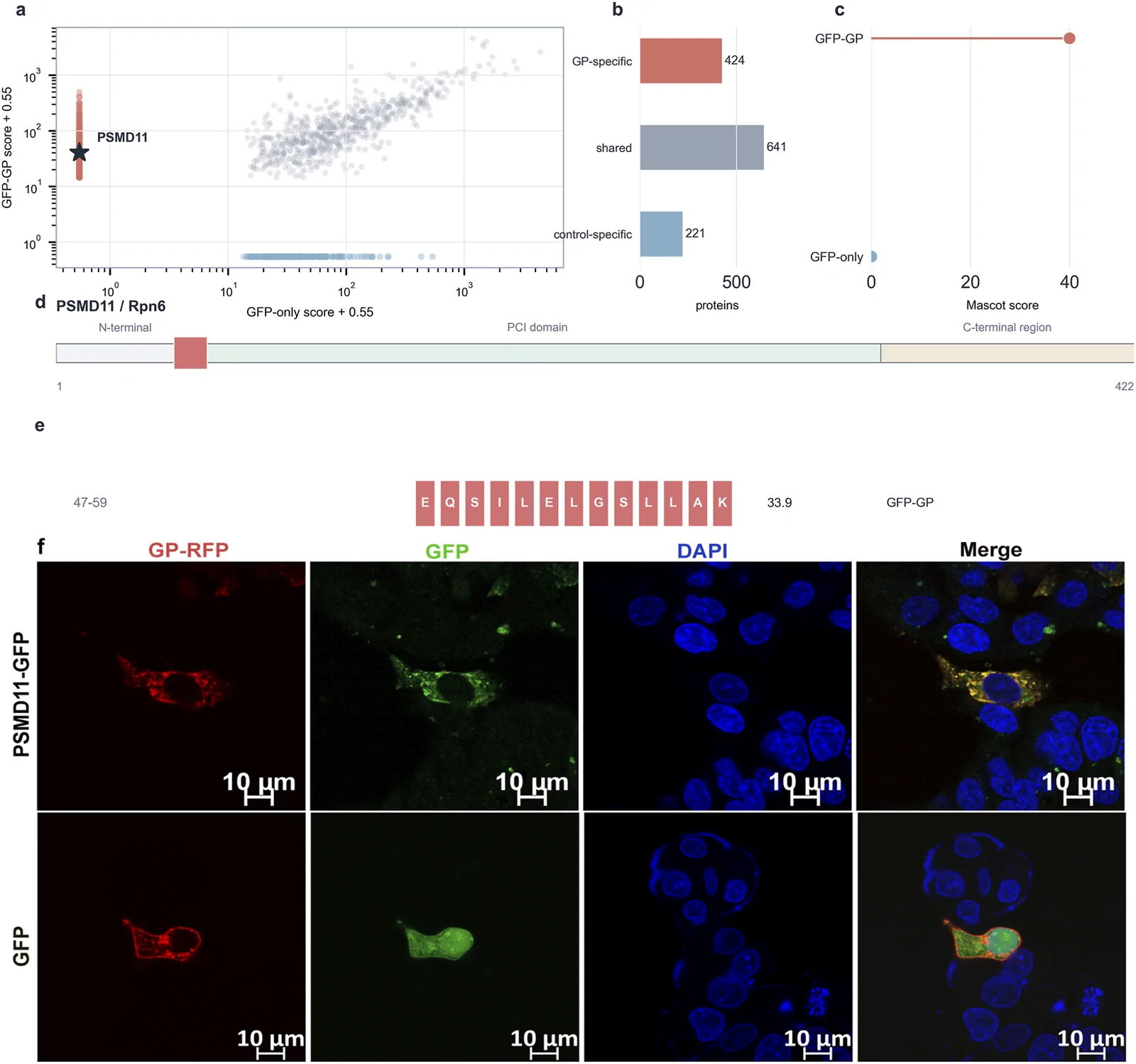
PSMD11/Rpn6 is identified as a GFP-GP pulldown-associated host candidate by Co-IP/MS and shows cellular spatial overlap with GP. **(a)** Mascot protein scores of UniProt accessions detected in GFP-GP pulldown and GFP-only control samples. Proteins detected only in GFP-GP were classified as GP-specific candidates, proteins detected in both groups were classified as shared/non-specific proteins, and proteins detected only in GFP-only controls were classified as GFP/bead-associated background. **(b)** Distribution of GP-specific, shared and control-specific proteins, comprising 424, 641 and 221 proteins, respectively. **(c)** Mass spectrometry evidence summary for PSMD11/Rpn6. **(d)** Schematic representation of PSMD11/Rpn6 protein length and peptide localization. **(e)** The PSMD11/Rpn6 peptide EQSILELGSLLAK maps to residues 47–59 and has a Mascot ion score of 33.9. **(f)** Confocal images of HeLa cells co-expressing GP-RFP (red) with PSMD11-GFP or GFP-alone control (green), with DAPI nuclear staining (blue). Scale bars, 10 μm.

To provide orthogonal cellular context for this nomination, we examined the spatial distribution of GP-RFP and PSMD11-GFP by confocal imaging. GP-RFP and PSMD11-GFP showed prominent spatial overlap in cytoplasmic and perinuclear regions, whereas the GFP-alone control did not reproduce this concentrated overlap pattern (Figure 2f). This observation places the two proteins in shared intracellular compartments and adds an orthogonal cellular evidence layer to the proteomic nomination of PSMD11/Rpn6 as a GP-associated host candidate.

### 3.2 PSMD11/Rpn6-centred KG separates contextual candidates from TransE-only reserves

The knowledge-graph layer converted the PSMD11/Rpn6 pulldown signal into an interpretable candidate queue in which every nomination is traceable to a named evidence component. The graph projection placed PSMD11/Rpn6 within a heterogeneous biomedical neighbourhood spanning proteins, compounds, viral-context terms, pathways and literature-derived evidence nodes (Figure 3a). Relation and source summaries confirmed that this neighbourhood draws on multiple independent evidence classes, including proteostasis-relevant relations, viral host context, DRKG-derived edges, curated literature, the MS seed and manually audited triples (Figure 3b,c). Known PSMD11/Rpn6 probes, direct PSMD11/Rpn6 evidence compounds and classical proteasome inhibitors were held as audit references, which keeps the exploratory ranking from collapsing onto already-characterized chemistry.

**FIGURE 3:**
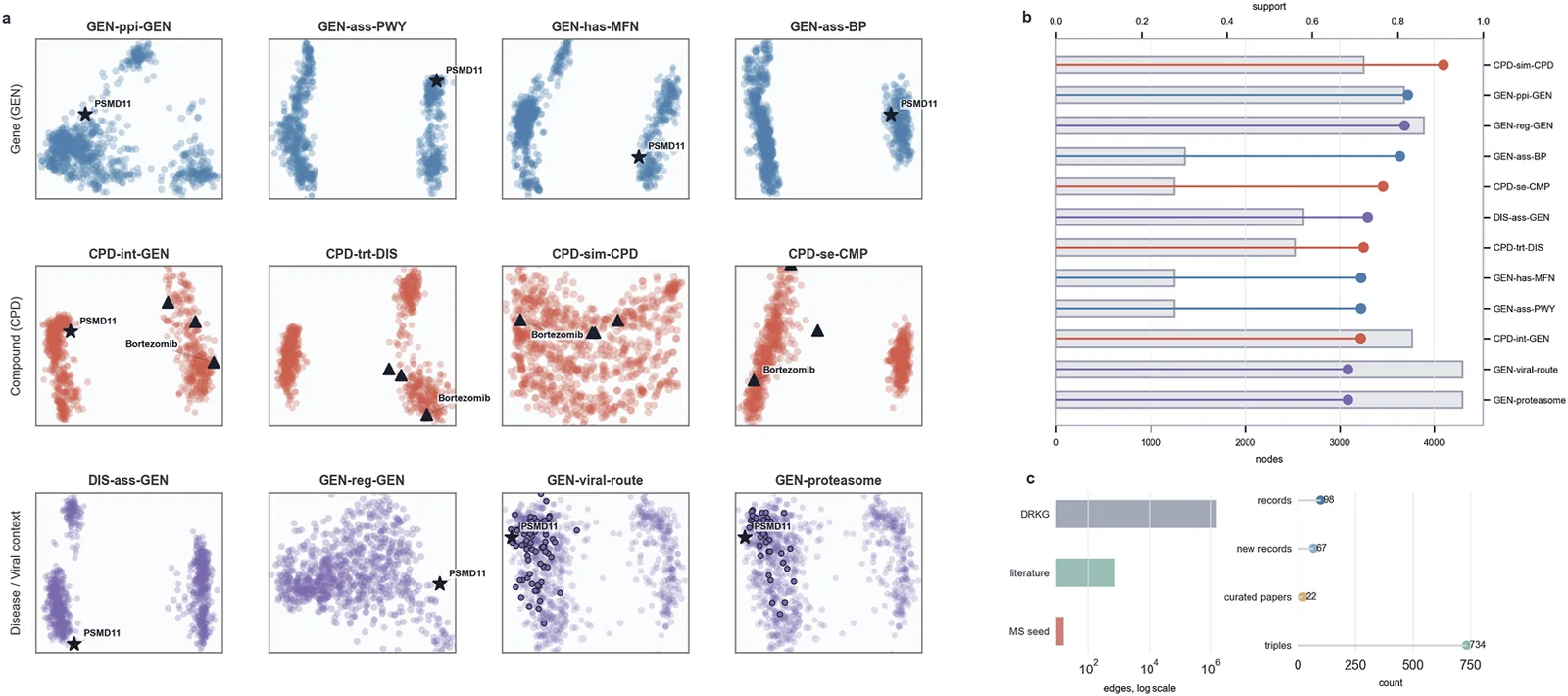
Literature evidence and heterogeneous biomedical KG construction centred on PSMD11/Rpn6. **(a)** Relation-specific 2D projections. Star, PSMD11/Rpn6; triangles, representative control compounds. Blue, red and purple denote gene-, compound- and disease/viral-context relations. GEN-viral-route and GEN-proteasome share the same gene–gene coordinates; darker outlined points mark the viral-host and PSMD11-centred proteasome neighbourhoods, respectively. **(b)** Node counts (grey bars) and support (lollipops) by relation type. **(c)** Source composition of DRKG, curated literature and MS-seed edges (left, log scale) and the corresponding record-level counts (right).

Figure 4 traces the full path from query to queue. A PSMD11/Rpn6-centred query was situated in a heterogeneous EBOV-GP/host-proteostasis schema (Figure 4a), and candidate compound links were then scored on five declared components: TransE-supported graph completion, PSMD11-neighbourhood support, proteasome-module membership, shared-anchor transfer and a target-count penalty (Figure 4b). The two-tier display separates context-supported candidates from reserve-only TransE hits, so contextual evidence governs nomination while reserve hits remain visible for later exploration (Figure 4c), and the evidence decomposition reports which component carried each candidate before ML and ADMET profiling (Figure 4d). The result is a KG top-20 queue delivered to ligand-only ML filtering (Supplementary Table S1), with each entry annotated by its supporting mechanism: CHEMBL1410015 led the queue on a USP14-linked transfer signal combined with low target_count, while Everolimus, Betamethasone and Pitavastatin entered as mTOR/proteostasis, steroid and metabolic-transfer hypotheses respectively. Weight-sensitivity analysis established that the queue is a property of the evidence architecture rather than of one parameter setting. Equal component weighting retained all 12 tier-1 candidates at near-identical ordering (Spearman rho = 0.998 versus baseline), and the tier-gated top-12 set was preserved across every predefined, single-component and Dirichlet perturbation scenario tested (Supplementary Figure S1; Supplementary Table S6).

**FIGURE 4:**
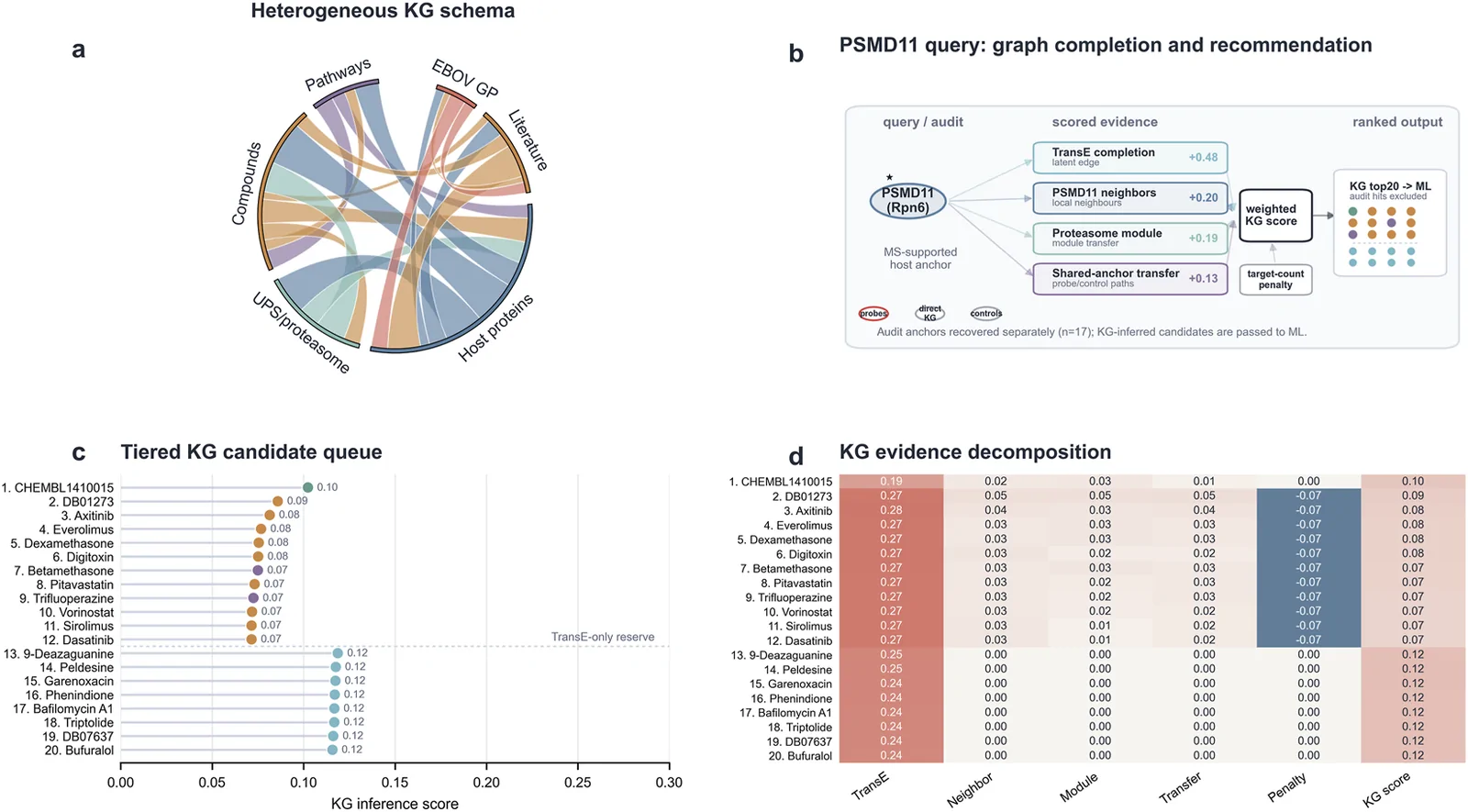
KG inference converts the PSMD11/Rpn6 anchor into an interpretable top20 candidate queue. **(a)** Heterogeneous KG schema linking EBOV GP, literature evidence, host proteins, UPS/proteasome context, compounds and pathways. **(b)** PSMD11/Rpn6-centred graph-completion query, showing TransE completion, PSMD11-neighbourhood support, proteasome-module evidence, shared-anchor transfer and target-count penalty before candidates are passed to ML. Published Rpn6 probes, direct PSMD11/Rpn6 KG relations and classical proteasome inhibitors are recovered as audit anchors rather than discovery hits. **(c)** Rule-tiered KG top20 queue, separating context-supported ranks 1–12 from TransE-only reserve ranks 13–20. **(d)** Evidence decomposition across the top20 queue before ligand-only ML and ADMET profiling; cell labels report the displayed contribution values.

The target-count penalty proved to be the decisive term, and its behaviour demonstrates why the queue is tier-gated. Removing the penalty left the tier-gated ranking essentially intact (rho = 0.901) but inverted the score-only ranking (rho = −0.489), because the penalty is what prevents highly promiscuous compounds from occupying the top of a discovery-oriented queue. Staged tier rules therefore govern candidate nomination, and this design gives the queue a stability that raw score ordering does not possess.

### 3.3 Ligand-only filtering adds activity-space prioritization without duplicating KG rank

Ligand-only machine learning contributed an activity-space axis that is orthogonal to the knowledge graph by construction: the KG encodes PSMD11-side network context, whereas the ML layer scores relative activity tendency within the available bioactivity feature space. Combining two independent axes, rather than re-weighting a single one, is what allows the workflow to distinguish network-supported from chemistry-supported candidates. Model benchmarking selected ExtraTrees and quantified how far each endpoint head could be trusted (Figure 5a,b). The deployed models reached AUC values of 0.912 for Ki, 0.845 for IC50 and 0.775 for Kd. This endpoint-resolved performance profile directly determined the scoring rule: rather than averaging endpoints equally, the composite score was weighted toward Ki, the head with the strongest discrimination. The deployment panel makes the weighted Ki, IC50 and Kd contributions explicit for every candidate entering ADMET profiling (Figure 5c).

**FIGURE 5:**
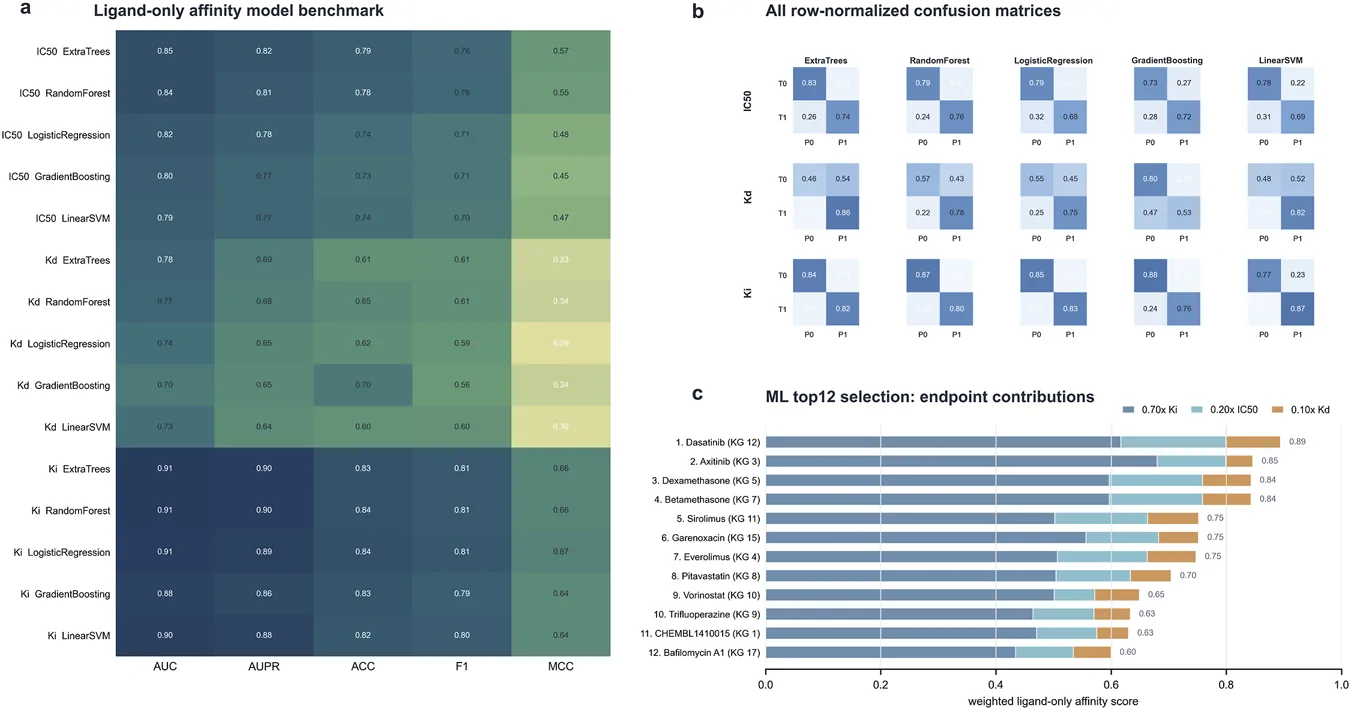
Ligand-only machine-learning models refine KG top20 candidates into an ML top12 set. **(a)** Performance comparison of IC50, Kd and Ki endpoint heads across Extra Trees, Random Forest, Logistic Regression, Gradient Boosting and Linear SVM models. Metrics include AUC, AUPR, accuracy, F1 score and Matthews correlation coefficient. **(b)** Row-normalized confusion matrices across all endpoint-model combinations, showing true-class-normalized T0/T1 classification behavior. **(c)** Deployment of the selected ExtraTrees endpoint heads as a weighted contribution score. Stacked bars show the Ki, IC50 and Kd contributions used to generate the ML top12 set for ADMET profiling, with the integrated ML affinity score defined as 0.70x Ki + 0.20x IC50 + 0.10x Kd.

The resulting ML top-12 was headed by Dasatinib, Axitinib, Dexamethasone and Betamethasone. CHEMBL1410015 moved from KG rank 1 to ML rank 11 and Bafilomycin A1 ranked 12th (Supplementary Table S2). This divergence is the informative output of the layer: candidates that rank highly on both axes carry independent support, while candidates that rank highly on one axis are retained for the specific evidence they represent. Endpoint-weight sensitivity confirmed the stability of the pool. Every weighting scenario tested returned the identical ML top-12 membership, and equal Ki/IC50/Kd weighting preserved four of the five baseline top-five candidates with high rank concordance (rho = 0.839). Candidate selection therefore depends on the composite evidence rather than on the specific endpoint weights, with fine ordering inside the top five sensitive to weighting as expected (Supplementary Figure S2; Supplementary Table S6).

An applicability-domain audit then assigned each candidate an explicit confidence tier, so that ML scores are used only within their supported chemical space. Four of the 12 candidates were inside or well supported, six borderline or mixed and two extrapolative. Among the five compounds carried forward, Betamethasone fell inside the well-supported tier, which is the basis for treating it as the ML-supported steroid representative. Everolimus, Pitavastatin and CHEMBL1410015 were borderline or mixed. Bafilomycin A1 sits outside the training chemistry entirely, with a maximum ECFP4 Tanimoto similarity of 0.20–0.24 against any training ligand (Supplementary Figure S3; Supplementary Table S7); it is accordingly carried as a pathway-oriented comparator on V-ATPase and lysosomal-proteostasis grounds, and its priority does not rest on the ML axis. This audit is what makes the ML layer usable downstream: each of the five structural follow-up candidates enters the next stage with a defined evidence source and a defined domain of validity.

### 3.4 ADMET profiling redirects broad-liability compounds away from structural follow-up

ADMET profiling changed the interpretation of the ML top12 by separating predicted activity tendency from practical follow-up value. After reordering by local ADMET burden, the liability heatmap showed that several high-ML candidates, including Dasatinib, Axitinib and Trifluoperazine, carried broad predicted liabilities across CYP interaction, DILI, genotoxicity, organ-toxicity and assay-interference dimensions (Figure 6a). The radar plot summarized how the five-candidate docked set differed from the full ML top12 and the red-flag group (Figure 6b), while the overall burden panel made the practical triage visible (Figure 6c). Dexamethasone and Betamethasone had the lowest local overall_admet_risk_proxy values in this set (0.413 each), followed by Bafilomycin A1, Everolimus, Sirolimus, Pitavastatin and CHEMBL1410015. By contrast, Dasatinib, Axitinib and Trifluoperazine retained strong ML ranks but carried multidimensional risk patterns, so they were reassigned to comparator or control roles (Supplementary Table S3). ADMET profiling served as an additional pharmacological prioritization layer by evaluating predicted liabilities and defining a feasible candidate space for subsequent structural compatibility assessment. The BBB permeability endpoint further characterized the prioritized compounds according to their predicted anatomical distribution. Dexamethasone, Betamethasone, Vorinostat, Trifluoperazine, and CHEMBL1410015 were predicted to exhibit CNS-penetrant properties, whereas Dasatinib, Axitinib, Sirolimus, Garenoxacin, Everolimus, Pitavastatin, and Bafilomycin A1 showed limited predicted BBB permeability. Given that EBOV infection involves systemic replication and may involve CNS-associated manifestations, BBB permeability provides an important pharmacokinetic context for evaluating the potential application scope of different candidates. Therefore, compounds with distinct predicted compartment profiles were retained for downstream characterization, allowing compartment distribution to be considered as an additional pharmacological variable. The structural assessment set included CNS-predicted candidates (CHEMBL1410015 and Betamethasone) and peripherally restricted candidates (Pitavastatin, Bafilomycin A1, and Everolimus), providing candidate diversity for subsequent evaluation of their biological and pharmacological properties (Supplementary Figure S4; Supplementary Table S7).

**FIGURE 6:**
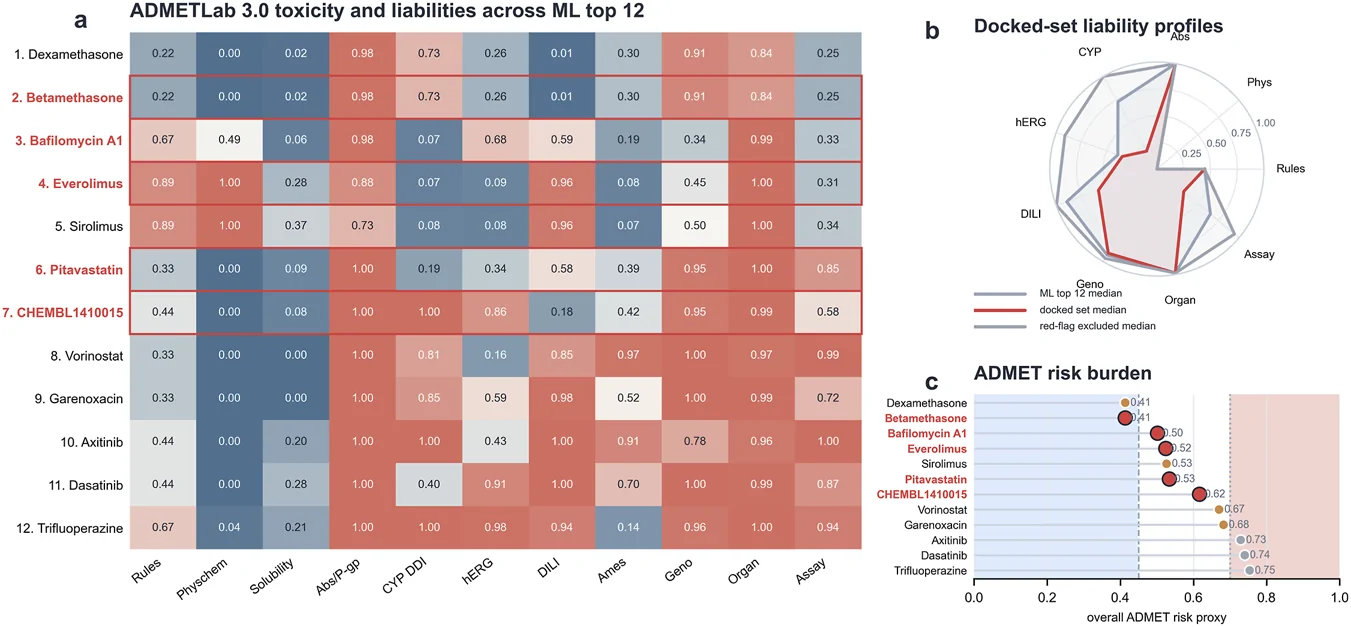
ADMET risk profiling supports a mechanism-diverse structural follow-up shortlist. **(a)** ADMET toxicity/liability heatmap of ML top12 candidates reordered by local ADMET risk burden; heatmap cell labels show the displayed liability values. Red outlines mark the five candidates selected for docking: Betamethasone, Bafilomycin A1, Everolimus, Pitavastatin and CHEMBL1410015. Higher values indicate greater locally derived risk proxy burden. **(b)** Liability radar comparing the median risk profile of the ML top12, the five-candidate docked set and the red-flag excluded group. **(c)** Overall ADMET risk burden and candidate stratification after ADMET reordering. Docked candidates are indicated with red markers. The blue, neutral and muted-red background bands denote local low, intermediate and high risk zones used only for this cohort (<0.45, 0.45–0.70 and >0.70). The docking shortlist was not selected by ADMET rank alone or by a single composite KG-ML-ADMET score.

The structural follow-up set was assembled by staged filtering across all four evidence layers. KG inference generated a PSMD11/Rpn6-centred top20 queue, ligand-only ML reduced this set to an activity-supported top12, and ADMET profiling removed or downgraded candidates with broad predicted liabilities. The final five were then chosen from the ADMET-filtered pool by four practical criteria: acceptable within-cohort ADMET burden, at least one upstream support signal from KG or ML, non-redundant chemistry/mechanism and suitability for interface-proximal docking. This rule-based triage explains why Betamethasone was retained as the steroid representative while Dexamethasone was not advanced independently, and why Everolimus represented the rapalog/mTOR space while Sirolimus was kept as a reference. It also preserved Bafilomycin A1 despite its reserve-tier KG position because it provided a V-ATPase/autophagy-proteostasis comparator with low target_count and acceptable within-cohort ADMET burden. Pitavastatin was retained as a statin/metabolic-transfer hypothesis and CHEMBL1410015 as the KG-driven USP14-linked exploratory signal. The final docking shortlist was therefore Betamethasone, Bafilomycin A1, Everolimus, Pitavastatin and CHEMBL1410015. Candidate selection integrated mechanism, predicted liability, chemical class and experimental interpretability, so each shortlisted compound carries a distinct and traceable evidence basis. The final structural follow-up shortlist and its role assignments are provided in Supplementary Table S4.

### 3.5 Interface-focused docking supports structural accommodation of shortlisted candidates

Interface-focused docking served as the final triage layer of the workflow, converting the five prioritized compounds into a ranked set of regional accommodation hypotheses with quantified confidence bounds. Every reported value is paired with a protocol audit, so the structural conclusions rest on reproducibility, spatial context and library calibration rather than on any individual number. The predicted PSMD11/Rpn6-EBOV GP complex supplied the PSMD11/Rpn6-side GP-contacting footprint that defined pocket selection and docking-box placement (Figure 7a). Everolimus produced the strongest Vina mode-1 value in the pre-specified five-candidate set (−7.2 kcal/mol), followed by Bafilomycin A1 (−6.3 kcal/mol), Betamethasone (−6.1 kcal/mol), Pitavastatin (−5.7 kcal/mol) and CHEMBL1410015 (−5.3 kcal/mol). Figure 7 shows representative poses for Betamethasone, CHEMBL1410015 and Bafilomycin A1, and Table 1 summarizes all five structural follow-up candidates (Supplementary Table S5). Proteasome-inhibitor audit ligands present in the docking output files served as protocol references and are reported separately.

**FIGURE 7:**
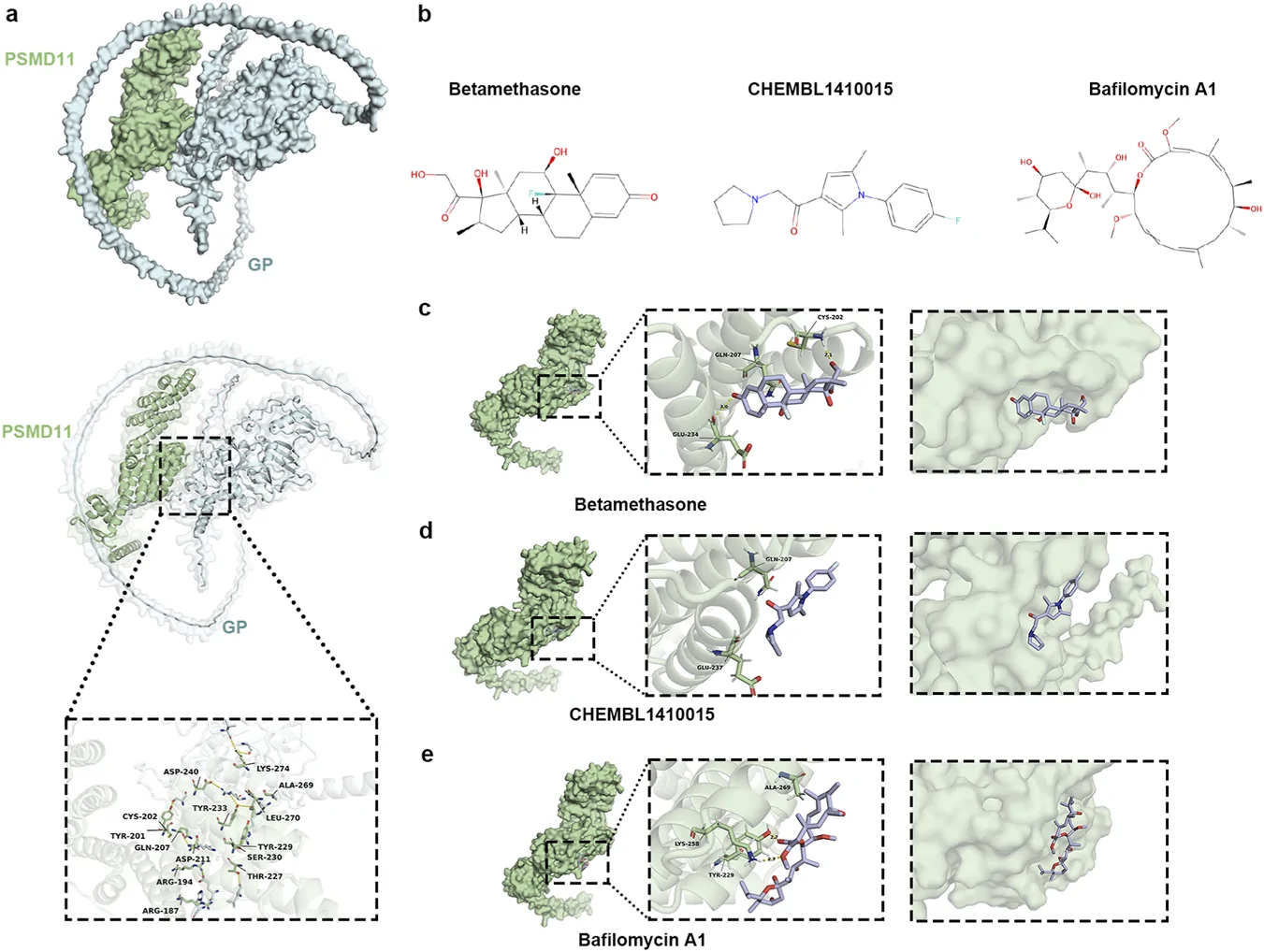
Preliminary interface docking of representative structural follow-up candidates. **(a)** Predicted PSMD11/Rpn6-EBOV GP complex used to define the GP-contacting footprint on the PSMD11/Rpn6 side. PSMD11/Rpn6 is shown in green and EBOV GP is shown in light blue. The enlarged interface view highlights PSMD11/Rpn6 residues located within the predicted protein-protein interaction footprint. **(b–e)** show representative structures and poses for three of the five shortlisted candidates: Betamethasone, CHEMBL1410015 and Bafilomycin A1. For **(c–e)**, the left subpanel shows the ligand position within the PSMD11/Rpn6 structure, the middle subpanel shows residue-level interactions near the predicted GP-contacting interface, and the right subpanel shows the ligand accommodated within the local surface pocket. Candidate molecules are shown as stick models, and yellow dashed lines indicate predicted hydrogen bonds. Docking affinities for all five pre-specified structural follow-up candidates are reported in Table 1.

**TABLE 1:** Docking affinity summary for structural follow-up candidates.

Candidate	Vina affinity (kcal/mol)
Everolimus	−7.2
Bafilomycin A1	−6.3
Betamethasone	−6.1
Pitavastatin	−5.7
CHEMBL1410015	−5.3

Sampling behaviour was characterized directly. Five-seed re-docking reproduced the reported values closely for the three conformationally restricted candidates (maximum standard deviation 0.05 kcal/mol; maximum deviation from the reported value 0.13 kcal/mol). Flexible and macrocyclic ligands showed a wider seed-dependent range, reaching 0.35 kcal/mol and 0.76 kcal/mol for Everolimus, reported alongside the best sampled pose. Raising exhaustiveness from 8 to 32 changed the best score by at most 0.03 kcal/mol, establishing that the search had converged and that the differences among candidates are not sampling artefacts.

Whole-receptor docking mapped the spatial context of the selected region and located further accommodating surface patches, with score differences of 0.37–1.86 kcal/mol relative to the interface-proximal search. The interface-proximal model is therefore defined as a local structural hypothesis suitable for targeted experimental testing, and the whole-receptor map identifies the alternative regions that the same experiments will resolve. Calibration against the matched 59-compound KG-scored pool (median −5.68 kcal/mol; range −7.9 to −2.9) converts these values into an actionable hierarchy. Everolimus ranked within the top fifth percentile of the pool, Bafilomycin A1 within the top 20th and Betamethasone within the top 29th. The Everolimus result is consistent with its large multi-functional macrolide scaffold, which engages an extended contact surface within the pocket, and this position within a workflow-internal reference distribution provides the basis for ordering experimental follow-up.

Macrocycle ring treatment, the dominant protocol variable for two of the five candidates, was audited explicitly. The archived torsion trees contain 11 active torsions for Bafilomycin A1 and 12 for Everolimus, against 22 and 33 under macrocycle-aware preparation, so both were docked with rigid rings from a single input conformer. A scale-matched control docking 20 ETKDGv3 ring conformers of each macrocycle with the ring held rigid spanned 1.35 kcal/mol for Bafilomycin A1 and 1.93 kcal/mol for Everolimus; input conformer selection therefore moves macrocycle scores by more than 1 kcal/mol, and both values are reported with this bound attached.

Together these controls deliver a reproducible, convergence-verified and library-calibrated structural hierarchy in which each candidate carries an explicit uncertainty bound, defining the regional accommodation hypotheses that direct experimental testing will address (Supplementary Figures S5-S7; Supplementary Tables S8-S11).

## 4 Discussion

Host-directed antiviral discovery has produced extensive catalogues of virus-associated host factors, yet converting these catalogues into testable chemical hypotheses remains the rate-limiting step. Computational repurposing addresses the complementary problem, exploring chemical space rapidly while lacking experimentally derived biological anchors. The present work connects the two. GFP-GP pulldown Co-IP/MS identified PSMD11/Rpn6 as a GP-associated proteostasis component, and knowledge-graph inference, ligand-based machine learning, ADMET assessment and interface-oriented docking were then applied around that single anchor to generate a compact set of prioritized compounds.

PSMD11/Rpn6 is a regulatory-particle subunit of the 26S proteasome that governs proteasome assembly and substrate-processing capacity. EBOV GP biosynthesis imposes a heavy load on host quality control through extensive folding, dense glycosylation and multi-compartment trafficking, so enrichment of a proteasome assembly factor in the GP pulldown relative to the matched GFP-only control links GP expression to proteostasis regulation. Co-IP/MS reports co-capture within a complex, which is consistent with direct binding, indirect recruitment, shared localization or a GP-induced shift in quality-control machinery. PSMD11/Rpn6 therefore functions here as a biologically motivated anchor for compound discovery. Among the 424 proteins detected only in the GP condition, it was selected on canonical peptide support, a defined role in the regulatory particle, published chemical tractability and the mechanistic link between proteostasis and viral glycoprotein maturation.

The resulting candidates carry distinct evidence signatures rather than positions on a single scale. The knowledge graph supplies biological context around PSMD11/Rpn6, machine learning estimates activity tendency within known chemical-biological space, ADMET profiling addresses pharmacological feasibility, and docking supplies structural compatibility hypotheses. Betamethasone emerged as a steroid-associated representative combining favourable predicted activity with acceptable ADMET properties. Bafilomycin A1 entered as a pathway-oriented comparator through its established relationship with V-ATPase, lysosomal regulation and proteostasis. Everolimus and Pitavastatin represent mTOR-associated and metabolic-regulatory mechanisms. CHEMBL1410015 is a knowledge-graph-driven exploratory candidate supported by a USP14-linked inference signal and a low target-count penalty. Reading the output by mechanistic role preserves more biological information than a single composite score, since compounds entering through different evidence layers demand different validation experiments.

Sensitivity analysis of the scoring framework supports this prioritization. Equal component weighting, single-component perturbation, variation of the target-count penalty and random Dirichlet weight sampling all reproduced consistent candidate rankings (Supplementary Figure S1; Supplementary Table S6). Candidate selection thus reflects the combined contribution of several evidence dimensions rather than one predefined weighting configuration.

The scope of the present evidence is defined by three features of the study design. The Co-IP/MS analysis used one pulldown sample per condition, so the GP-associated signal establishes a candidate rather than a quantified interaction. Support for PSMD11/Rpn6 rests on proteomic enrichment and cellular localization, leaving direct molecular contact and interface geometry open. The HEK293 GFP-GP overexpression system offers a controlled discovery platform in which replication dynamics, endogenous GP levels and host responses differ from those of EBOV infection.

Structural uncertainty likewise shapes what the computational analyses can claim. The predicted GP-PSMD11/Rpn6 complex carries low interface confidence, and blind docking distributed poses broadly across the receptor without favouring the predicted interface. Under these conditions, trajectory-based and physics-based rescoring methods characterize the behaviour of one selected model and report free energies for an arrangement that structural evidence does not yet single out. Their interpretive value depends on an experimentally supported binding site and a constrained pose, which cross-linking mass spectrometry, hydrogen-deuterium exchange mass spectrometry, truncation analysis and site-directed mutagenesis are positioned to provide. We are therefore pursuing interface characterization as the step that makes molecular dynamics and MM-GBSA/MM-PBSA analysis mechanistically informative.

Our validation programme follows the order in which this evidence must be established. We are first establishing the GP-PSMD11/Rpn6 association through reciprocal co-immunoprecipitation, endogenous-level detection and interface mapping. Direct compound engagement with PSMD11/Rpn6 will then be measured by orthogonal biophysical methods together with cellular target-engagement assays. Functional experiments, including competition assays, genetic perturbation and rescue, will test whether the candidates modulate GP-associated processes through PSMD11/Rpn6. Antiviral activity will be assessed in GP-pseudotyped entry systems and, where the biosafety context permits, authentic EBOV infection models, with pathway-matched counterscreens and cytotoxicity profiling to separate host-targeted mechanisms from the established pharmacological activities of the repurposed compounds.

Taken together, these results identify PSMD11/Rpn6-associated proteostasis as a plausible and experimentally addressable entry point for EBOV GP-focused host-directed discovery. Integrating proteomic evidence, knowledge-guided inference, machine learning prioritization and structural analysis within one transparent framework offers a scalable route from host-factor signals to focused therapeutic hypotheses.

## 5 Conclusion

This study provides a host-anchored strategy for translating an EBOV GP-associated proteomics signal into prioritized, experimentally tractable small-molecule hypotheses. GFP-GP pulldown Co-IP/MS nominated PSMD11/Rpn6 as a GP-associated proteostasis candidate, and this anchor was used to organize knowledge-graph inference, ligand-only machine learning, ADMET risk stratification and interface-oriented docking into a coherent triage workflow. Betamethasone, Bafilomycin A1, Everolimus, Pitavastatin and CHEMBL1410015 constitute the rule-selected structural follow-up set, each carrying a defined level of network support, predicted activity tendency, risk burden and structural accommodation. This distinction is essential because host-directed antiviral discovery requires not only computational ranking but also explicit control of mechanism, toxicity and assay interpretability. The framework advances a reproducible path from AP-MS-derived host-candidate discovery to mechanism-aware molecular triage. It integrates a GP-associated experimental anchor, stable KG and ML candidate pools, ADMET-informed selection and a calibration-aware structural screen to deliver a chemically diverse, experiment-ready shortlist.

## Data Availability

The original contributions presented in the study are publicly available. This data can be found here: ProteomeXchange/PRIDE accession PXD084580 (https://www.ebi.ac.uk/pride/archive/projects/PXD084580).
