# Supplementary material for: Knowledge graph and machine learning-guided virtual screening prioritizes putative blockers of the PSMD11/Rpn6-EBOV glycoprotein interface
Source: Front Pharmacol. 2026 Sep 29;17:1910440. doi: 10.3389/fphar.2026.1910440 (PMC13657989; doi:10.3389/fphar.2026.1910440)
Supplement: Supplementary file 1 [file Supplementaryfile1.docx]

**Supplementary Figures and Tables**

*Supplementary Figs. S1-S4 and Supplementary Tables S6-S7 report the knowledge-graph and machine-learning sensitivity analyses, the ligand-only ML applicability-domain audit and the ADMET blood-brain barrier endpoint. Supplementary Figs. S5-S7 and Supplementary Tables S8-S11 report the docking control experiments performed on the archived AutoDock Vina input files.*

**Supplementary Fig. S1**

Supplementary Fig. S1. Knowledge-graph score-weight robustness under Dirichlet perturbation. Points show the median tier-gated rank; bars show the 5th-95th percentile interval. Marker size reflects top-five frequency; shading marks the top-five and top-12 zones. Equal component weights retained all 12 tier-1 candidates (Spearman rho = 0.998 versus baseline).

**Supplementary Fig. S2**

Supplementary Fig. S2. Ligand-only machine-learning endpoint-weight robustness. Rank intervals are shown under Dirichlet perturbations around the manuscript endpoint weights (0.70 Ki / 0.20 IC50 / 0.10 Kd) and under a broader uninformative prior. The ML top-12 pool was retained in every scenario; equal endpoint weights retained four of the five baseline top-five candidates (rho = 0.839).

**Supplementary Fig. S3**

Supplementary Fig. S3. Candidate-level ligand-only ML applicability-domain audit. Each candidate is summarized by endpoint-specific ECFP4 nearest-neighbour Tanimoto support against the IC50, Kd and Ki training ligands, together with its overall applicability-domain call. Four candidates are inside or well supported, six borderline or mixed, and two extrapolative. The two extrapolative candidates are Garenoxacin and Bafilomycin A1 (nearest-neighbour Tanimoto 0.20-0.25); of these, only Bafilomycin A1 is in the five-compound structural follow-up set.

**Supplementary Fig. S4**

Supplementary Fig. S4. ADMETLab BBB penetration probability across the ML top-12. (a) Candidate-level predicted probabilities, with the 0.7 high-penetration call marked. Bars are coloured by predicted compartment and by membership of the structural follow-up set (asterisk). (b) Compartment coverage of the five structural follow-up candidates: CHEMBL1410015 and Betamethasone are predicted CNS-penetrant, and Pitavastatin, Bafilomycin A1 and Everolimus peripherally restricted. Because EBOV persists in immune-privileged CNS sites, the endpoint is reported as compartment coverage informing assay and in vivo design rather than applied as a selection filter.

**Supplementary Fig. S5**

Supplementary Fig. S5. Docking reproducibility and blind-docking site-preference control. (a) Each archived run re-docked under five random seeds with the archived configuration; red dashes mark the value reported in the manuscript. The maximum standard deviation across seeds was 0.35 kcal/mol and the maximum deviation from the reported value 0.76 kcal/mol, so the published numbers are stable sampling outputs. (b) Best score in the archived interface box versus a whole-receptor blind search (108 x 68 x 84 A), five seeds each. For 7 of 7 ligands the blind search found a score at least as good as the interface search (largest gain 1.9 kcal/mol), indicating that the predicted interface is not a preferred site.

**Supplementary Fig. S6**

Supplementary Fig. S6. Macrocycle sampling protocol comparison for the two macrocyclic candidates. (a) Active torsions in the docking tree. The archived preparation froze all macrocyclic ring bonds; macrocycle-aware preparation with glue pseudo-atoms raises the sampled degrees of freedom by up to 21 torsions. (b) Best Vina score under the rigid-ring preparation, the macrocycle-aware preparation and a 20-member ETKDGv3 ring-conformer ensemble, against the value reported in the manuscript. Note that glue-atom (macrocycle-aware) scores are not on the same scale as rigid-ring scores, because the glue formalism adds ring-closure terms to the Vina scoring function; the ring-conformer ensemble keeps the ring rigid within each conformer and is therefore the directly comparable control. The panel documents the protocol dependence of the reported numbers, not a corrected affinity.

**Supplementary Fig. S7**

Supplementary Fig. S7. Score-distribution baseline for the archived interface box. The manuscript's KG-scored candidate pool (59 ligands from the PSMD11-side target-transfer and TransE-completion tiers, with published audit anchors and proteasome-inhibitor positive controls excluded) was docked into the identical box with identical settings. The library median is -5.68 kcal/mol and 19 of 59 compounds (32%) score at or below -6.0 kcal/mol. The five shortlisted compounds fall within this distribution rather than as outliers.

**Supplementary Fig. S8**

Supplementary Fig. S8. Original GFP pull-down gel image from the GFP-GP and GFP-only control experiment used for the pull-down/MS workflow. The arrow indicates GFP-GP. This image documents the original experimental gel material; it is not a quantitative replicate analysis or an LC-MS/MS instrument spectrum.

**Supplementary Tables**

**Supplementary Table S6**

Supplementary Table S6. Knowledge-graph and machine-learning score-weight sensitivity analysis. Spearman correlation against the baseline ranking, and top-five / top-12 overlap, for every predefined and perturbation scenario. Note the score-only column for the no-target-count-penalty scenario (rho = -0.489): the target-count penalty is the dominant term in the raw score, which is why candidate nomination is governed by the staged tier rules rather than by the raw KG score.

| **layer** | **scenario** | **scenario type** | **tiered spearman vs baseline** | **score only spearman vs baseline** | **top5 overlap** | **top12 overlap** |
| --- | --- | --- | --- | --- | --- | --- |
| KG | baseline_weights_penalty_1.0 | predefined | 1.000 | 1.000 | 5 | 12 |
| KG | equal_component_weights_penalty_1.0 | predefined | 0.998 | 0.944 | 5 | 12 |
| KG | baseline_weights_no_target_count_penalty | predefined | 0.901 | -0.489 | 4 | 12 |
| KG | baseline_weights_half_target_count_penalty | predefined | 0.901 | 0.824 | 4 | 12 |
| KG | baseline_weights_1.5x_target_count_penalty | predefined | 1.000 | 1.000 | 5 | 12 |
| KG | transe-completion_x0.5_renormalized | one_component_perturbation | 0.998 | 0.998 | 5 | 12 |
| KG | transe-completion_x0.75_renormalized | one_component_perturbation | 0.998 | 0.998 | 5 | 12 |
| KG | transe-completion_x1.25_renormalized | one_component_perturbation | 1.000 | 1.000 | 5 | 12 |
| KG | transe-completion_x1.5_renormalized | one_component_perturbation | 0.994 | 0.994 | 4 | 12 |
| KG | psmd11-neighbor_x0.5_renormalized | one_component_perturbation | 0.998 | 0.998 | 5 | 12 |
| KG | psmd11-neighbor_x0.75_renormalized | one_component_perturbation | 0.998 | 0.998 | 5 | 12 |
| KG | psmd11-neighbor_x1.25_renormalized | one_component_perturbation | 0.998 | 0.998 | 4 | 12 |
| KG | psmd11-neighbor_x1.5_renormalized | one_component_perturbation | 0.998 | 0.998 | 4 | 12 |
| KG | proteasome-module_x0.5_renormalized | one_component_perturbation | 0.994 | 0.994 | 4 | 12 |
| KG | proteasome-module_x0.75_renormalized | one_component_perturbation | 0.994 | 0.994 | 4 | 12 |
| KG | proteasome-module_x1.25_renormalized | one_component_perturbation | 0.998 | 0.998 | 5 | 12 |
| KG | proteasome-module_x1.5_renormalized | one_component_perturbation | 0.998 | 0.998 | 5 | 12 |
| KG | shared-anchor-transfer_x0.5_renormalized | one_component_perturbation | 0.998 | 0.998 | 4 | 12 |
| KG | shared-anchor-transfer_x0.75_renormalized | one_component_perturbation | 1.000 | 1.000 | 5 | 12 |
| KG | shared-anchor-transfer_x1.25_renormalized | one_component_perturbation | 0.998 | 0.998 | 5 | 12 |
| KG | shared-anchor-transfer_x1.5_renormalized | one_component_perturbation | 0.998 | 0.998 | 5 | 12 |
| ML | baseline_0.70Ki_0.20IC50_0.10Kd | predefined | 1.000 |  | 5 | 12 |
| ML | equal_endpoint_weights | predefined | 0.839 |  | 4 | 12 |
| ML | Ki_weight_x0.5_renormalized | one_endpoint_perturbation | 0.965 |  | 5 | 12 |
| ML | Ki_weight_x0.75_renormalized | one_endpoint_perturbation | 0.965 |  | 5 | 12 |
| ML | Ki_weight_x1.25_renormalized | one_endpoint_perturbation | 0.993 |  | 4 | 12 |
| ML | Ki_weight_x1.5_renormalized | one_endpoint_perturbation | 0.986 |  | 4 | 12 |
| ML | IC50_weight_x0.5_renormalized | one_endpoint_perturbation | 0.993 |  | 4 | 12 |
| ML | IC50_weight_x0.75_renormalized | one_endpoint_perturbation | 0.993 |  | 4 | 12 |
| ML | IC50_weight_x1.25_renormalized | one_endpoint_perturbation | 0.972 |  | 5 | 12 |
| ML | IC50_weight_x1.5_renormalized | one_endpoint_perturbation | 0.965 |  | 5 | 12 |
| ML | Kd_weight_x0.5_renormalized | one_endpoint_perturbation | 0.986 |  | 4 | 12 |
| ML | Kd_weight_x0.75_renormalized | one_endpoint_perturbation | 0.993 |  | 4 | 12 |
| ML | Kd_weight_x1.25_renormalized | one_endpoint_perturbation | 0.979 |  | 5 | 12 |
| ML | Kd_weight_x1.5_renormalized | one_endpoint_perturbation | 0.972 |  | 5 | 12 |

**Supplementary Table S7**

Supplementary Table S7. Candidate-level ligand-only ML applicability-domain calls and ADMET blood-brain barrier endpoint for the ML top-12 set, with structural follow-up roles.

| **compound id** | **drug name** | **ml rank** | **kg rank** | **overall applicability call** | **min max tanimoto** | **mean max tanimoto** | **mean top5 tanimoto** | **max abs descriptor robust z** | **BBB probability** | **BBB call** | **PAMPA endpoint** | **Pgp inhibitor probability** | **Pgp substrate probability** | **overall admet risk proxy** | **structural followup flag** | **structural followup role** | **chemotype** |
| --- | --- | --- | --- | --- | --- | --- | --- | --- | --- | --- | --- | --- | --- | --- | --- | --- | --- |
| Compound::DB01254 | Dasatinib | 1 | 12 | overall_inside_or_well_supported | 0.304 | 0.768 | 0.456 | 1.000 | 0.001 | low_predicted_BBB_penetration_probability | 0.007 | 0.866 | 0.040 | 0.740 | False | not selected for structural panel | other |
| Compound::DB08916 | Axitinib | 2 | 3 | overall_borderline_or_mixed_support | 0.265 | 0.510 | 0.348 | 1.009 | 0.059 | low_predicted_BBB_penetration_probability | 0.132 | 0.691 | 0.003 | 0.729 | False | not selected for structural panel | broad-target kinase inhibitor |
| Compound::DB01234 | Dexamethasone | 3 | 5 | overall_inside_or_well_supported | 1.000 | 1.000 | 0.404 | 1.432 | 0.907 | high_predicted_BBB_penetration_probability | 0.733 | 0.020 | 0.034 | 0.413 | False | not selected for structural panel | glucocorticoid steroid |
| Compound::DB00443 | Betamethasone | 4 | 7 | overall_inside_or_well_supported | 1.000 | 1.000 | 0.404 | 1.432 | 0.907 | high_predicted_BBB_penetration_probability | 0.733 | 0.020 | 0.034 | 0.413 | True | representative docking pose shown | glucocorticoid steroid |
| Compound::DB00877 | Sirolimus | 5 | 11 | overall_borderline_or_mixed_support | 0.375 | 0.693 | 0.315 | 2.977 | 0.000 | low_predicted_BBB_penetration_probability | 0.999 | 0.391 | 0.101 | 0.526 | False | not selected for structural panel | rapalog mTOR/proteostasis |
| Compound::DB06160 | Garenoxacin | 6 | 15 | overall_extrapolative_for_ligand_only_ml | 0.213 | 0.227 | 0.207 | 0.762 | 0.001 | low_predicted_BBB_penetration_probability | 0.980 | 0.000 | 0.002 | 0.682 | False | not selected for structural panel | fluoroquinolone |
| Compound::DB01590 | Everolimus | 7 | 4 | overall_borderline_or_mixed_support | 0.340 | 0.610 | 0.291 | 3.223 | 0.000 | low_predicted_BBB_penetration_probability | 0.981 | 0.850 | 0.025 | 0.524 | True | docked candidate; affinity table only | rapalog mTOR/proteostasis |
| Compound::DB08860 | Pitavastatin | 8 | 8 | overall_borderline_or_mixed_support | 0.260 | 0.292 | 0.252 | 0.772 | 0.162 | low_predicted_BBB_penetration_probability | 0.423 | 0.000 | 0.000 | 0.534 | True | docked candidate; affinity table only | statin/metabolic transfer |
| Compound::DB02546 | Vorinostat | 9 | 10 | overall_inside_or_well_supported | 0.622 | 0.874 | 0.532 | 0.750 | 0.871 | high_predicted_BBB_penetration_probability | 0.967 | 0.001 | 0.523 | 0.670 | False | not selected for structural panel | HDAC inhibitor |
| Compound::DB00831 | Trifluoperazine | 10 | 9 | overall_borderline_or_mixed_support | 0.302 | 0.471 | 0.321 | 1.580 | 1.000 | high_predicted_BBB_penetration_probability | 0.000 | 1.000 | 1.000 | 0.754 | False | not selected for structural panel | phenothiazine |
| Compound::CHEMBL1410015 | CHEMBL1410015 | 11 | 1 | overall_borderline_or_mixed_support | 0.260 | 0.281 | 0.259 | 1.271 | 1.000 | high_predicted_BBB_penetration_probability | 0.015 | 0.627 | 0.036 | 0.616 | True | representative docking pose shown | USP14-linked inferred small molecule |
| Compound::DB06733 | Bafilomycin A1 | 12 | 17 | overall_extrapolative_for_ligand_only_ml | 0.205 | 0.245 | 0.221 | 1.483 | 0.000 | low_predicted_BBB_penetration_probability | 0.981 | 0.005 | 0.922 | 0.502 | True | representative docking pose shown | V-ATPase/autophagy-proteostasis |

**Supplementary Table S8**

Supplementary Table S8. Docking grid specification and seed-replicate reproducibility. The complete archived configuration was: box centre (-11.079, 5.480, -0.367) A; dimensions 40.14 x 25.64 x 43.68 A; volume 44952 A^3; grid spacing 0.375 A; exhaustiveness 8; energy_range 3 kcal/mol; num_modes 9; rigid receptor. This volume encloses 28.5% of receptor heavy atoms and 138 of 422 residues and exceeds the AutoDock Vina search-space warning threshold. Per-run seeds, per-mode scores and the contact-residue lists are in the accompanying CSV files.

| **compound** | **archived reported affinity** | **repro n seeds** | **repro mean** | **repro sd** | **repro min** | **repro max** | **repro range** | **max abs deviation from archived** | **exh32 n seeds** | **exh32 mean** | **exh32 best** | **exh32 gain vs exh8** | **ligand class** |
| --- | --- | --- | --- | --- | --- | --- | --- | --- | --- | --- | --- | --- | --- |
| Everolimus | -7.200 | 5 | -7.064 | 0.351 | -7.243 | -6.437 | 0.806 | 0.763 | 3 | -7.204 | -7.218 | 0.025 | flexible_or_macrocyclic |
| Bafilomycin A1 | -6.300 | 5 | -6.104 | 0.123 | -6.310 | -5.990 | 0.320 | 0.310 | 3 | -6.227 | -6.293 | 0.017 | flexible_or_macrocyclic |
| Betamethasone | -6.100 | 5 | -6.067 | 0.027 | -6.087 | -6.023 | 0.064 | 0.077 | 3 | -6.075 | -6.082 | 0.005 | rigid |
| Pitavastatin | -5.700 | 5 | -5.714 | 0.051 | -5.799 | -5.669 | 0.130 | 0.099 | 3 | -5.699 | -5.700 | 0.099 | rigid |
| CHEMBL1410015 | -5.300 | 5 | -5.402 | 0.028 | -5.426 | -5.359 | 0.067 | 0.126 | 3 | -5.386 | -5.426 | 0.000 | rigid |
| Carfilzomib | -6.400 | 5 | -6.445 | 0.154 | -6.686 | -6.288 | 0.398 | 0.286 | 3 | -6.292 | -6.460 | 0.226 | flexible_or_macrocyclic |
| Oprozomib | -6.200 | 5 | -6.003 | 0.119 | -6.161 | -5.894 | 0.267 | 0.306 | 3 | -6.162 | -6.188 | -0.027 | flexible_or_macrocyclic |

**Supplementary Table S9**

Supplementary Table S9. Blind-docking site-preference control. Best score over five seeds in the archived interface box compared with a whole-receptor search. A negative delta indicates that the blind search found a better score off-interface.

| **compound** | **interface best of 5 seeds** | **blind best of 5 seeds** | **blind mean** | **blind sd** | **delta blind minus interface** | **blind at least as good** |
| --- | --- | --- | --- | --- | --- | --- |
| Everolimus | -7.243 | -7.934 | -7.795 | 0.138 | -0.691 | True |
| Bafilomycin A1 | -6.310 | -6.676 | -6.316 | 0.356 | -0.366 | True |
| Betamethasone | -6.087 | -7.944 | -7.025 | 0.541 | -1.857 | True |
| Pitavastatin | -5.799 | -7.223 | -7.114 | 0.180 | -1.424 | True |
| CHEMBL1410015 | -5.426 | -7.008 | -6.291 | 0.498 | -1.582 | True |
| Carfilzomib | -6.686 | -7.723 | -7.117 | 0.390 | -1.037 | True |
| Oprozomib | -6.161 | -7.111 | -6.589 | 0.427 | -0.950 | True |

**Supplementary Table S10**

Supplementary Table S10. Macrocycle protocol comparison. Archived torsion counts are those present in the archived PDBQT torsion trees; macrocycle-aware counts are obtained with glue pseudo-atom ring opening. Glue-atom scores carry ring-closure penalty terms and are therefore not directly comparable in absolute value with the rigid-ring scores; the ring-conformer ensemble column is the scale-matched comparison. The three non-macrocyclic candidates were unaffected by the change of preparation and serve as an internal control.

| **compound** | **max ring size** | **archived reported affinity** | **archived torsions** | **macrocycle aware torsions** | **glue pseudo atoms** | **rigid ring best** | **macro glue best** | **macro glue mean** | **conformer ensemble n** | **conformer ensemble best** | **conformer ensemble mean** | **conformer ensemble worst** | **conformer ensemble spread** | **archived percentile in ensemble** | **delta glue minus rigid** |
| --- | --- | --- | --- | --- | --- | --- | --- | --- | --- | --- | --- | --- | --- | --- | --- |
| Bafilomycin A1 | 16 | -6.300 | 11 | 22 | 4 | -6.691 | -4.857 | -4.497 | 20 | -6.817 | -6.186 | -5.465 | 1.352 | 35.000 | 1.834 |
| Everolimus | 29 | -7.200 | 12 | 33 | 4 | -6.801 | -6.104 | -5.698 | 20 | -7.402 | -6.688 | -5.471 | 1.931 | 5.000 | 0.697 |

**Supplementary Table S11**

Supplementary Table S11. Compound-library score baseline. Percentile position of each shortlisted compound within the KG-scored candidate pool docked into the identical interface box. Audit anchors and proteasome-inhibitor positive controls are excluded from the pool, as are non-drug-like highly flexible chains (>32 active torsions, e.g. polyethoxylate detergents), for which Vina sampling does not converge; the exclusion list is provided as library_excluded_high_torsion.csv.

| **compound** | **best interface affinity** | **library n** | **library median** | **library mean** | **library sd** | **library best** | **n library at least as good** | **percentile within library** |
| --- | --- | --- | --- | --- | --- | --- | --- | --- |
| Everolimus | -7.243 | 59 | -5.677 | -5.658 | 0.907 | -7.895 | 3 | 5.100 |
| Bafilomycin A1 | -6.310 | 59 | -5.677 | -5.658 | 0.907 | -7.895 | 12 | 20.300 |
| Betamethasone | -6.087 | 59 | -5.677 | -5.658 | 0.907 | -7.895 | 17 | 28.800 |
| Pitavastatin | -5.799 | 59 | -5.677 | -5.658 | 0.907 | -7.895 | 24 | 40.700 |
| CHEMBL1410015 | -5.426 | 59 | -5.677 | -5.658 | 0.907 | -7.895 | 37 | 62.700 |
